# Multibacillary Leprosy in an Active Duty Military Member

**DOI:** 10.3201/eid2106.141666

**Published:** 2015-06

**Authors:** Catherine M. Berjohn, Christopher A. DuPlessis, Kathy Tieu, Ryan C. Maves

**Affiliations:** Naval Medical Center San Diego, San Diego, California, USA (C.M. Berjohn, R.C. Maves);; Naval Medical Research Center, Silver Spring, Maryland, USA (C.A. DuPlessis);; Fort Belvoir Community Hospital, Fort Belvoir, Virginia, USA (K. Tieu)

**Keywords:** leprosy, Hansen disease, multibacillary leprosy, Mycobacterium leprae, bacteria, tuberculosis and other mycobacteria, military personnel, epidemiology, diagnosis

**To the Editor:** Leprosy (Hansen disease) is caused by *Mycobacterium leprae*, an extremely slow-growing, intracellular, acid-fast bacillus with a typical incubation period >2–5 years, ranging up to 20 years. Anesthetic, thickened skin lesions and granulomatous inflammation in biopsy specimens are typical findings because direct visualization of the organism from biopsy specimens is unreliable (*1*).

The worldwide prevalence of leprosy is estimated to be <181,000 cases (annual incidence ≈220,000 new cases in recent years), of which 96% (173,760) occur in 14 countries (*2*). In the United States, ≈150 cases are reported annually, two thirds of which are associated with overseas exposure; the remainder are believed to be domestically acquired (*3*).

We report a case of multibacillary leprosy in a 44-year-old man, an active member of the US military, residing in southern California, USA, who had a 2-week history of fatigue and large, erythematous plaques on the extremities. He was born in the Philippines and resided there until immigrating to the United States at 23 years of age. He subsequently joined the US military and served as an administrator in clinical and microbiology research laboratories. He resided in California, Maryland, Japan, Egypt, Guam, and Indonesia. He also was deployed to Afghanistan and had vacationed in Laos, Cambodia, and Thailand. Family history was unremarkable. No household or other ill contacts were identified.

Initial evaluation showed a weakly positive antinuclear antibody titer, which prompted consideration of cutaneous lupus. Annular skin lesions subsequently developed on his face, limbs, and trunk (Figure, panel A). His fatigue persisted, and further rheumatologic evaluation did not show any unusual results. Dermatologic evaluation showed madarosis, thickening of the glabella, and 8 large annular plaques. Light touch sensation was impaired, but all lesions were hypersensitive to trauma.

**Figure:**
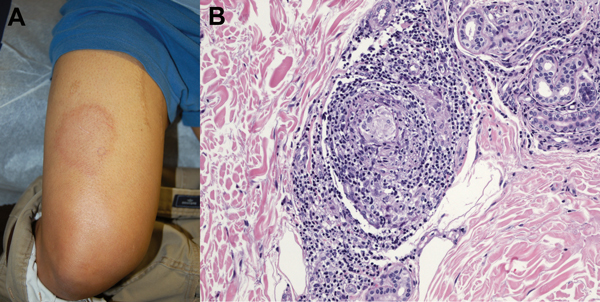
Physical examination and histopathologic manifestations of leprosy in a 44-year-old man in California, USA (active member of the US military). A) Large, annular, cutaneous plaque on the thigh. B) Skin biopsy specimen showing perineural lymphohistiocytic inflammation and non-necrotizing granulomas (hematoxylin and eosin stained, original magnification ×40).

A skin biopsy specimen showed perineural lymphohistiocytic inflammation and nonnecrotizing granulomata (Figure, panel B). Results of acid-fast and Grocott’s methenamine silver (fungal) staining were negative. A presumptive diagnosis of leprosy was made, and he was referred to the Division of Infectious Diseases at Naval Medical Center San Diego for subspecialty management. Dapsone and rifampin were given for multibacillary leprosy. Clofazamine was not available from the manufacturer at time of treatment. After 6 months of therapy, the patient’s lesions were less prominent, and cutaneous sensation had improved. A 2-year treatment course was completed and resulted in total resolution of cutaneous lesions. Residual anesthesia remained only over the right pinna. His course was without complication by either reversal reaction or erythema nodosum leprosum.

Understanding of the transmission of *M. leprae* has been impeded by difficulty cultivating the organism in vitro. Transmission is believed to occur by prolonged exposure to nasal secretions of patients with high bacillary loads. Infection might also result from exposure to cutaneous lesions or animal reservoirs, such as 9-banded armadillos (*3*).

Leprosy has been linked to defects in cell-mediated immunity. Milder disease has been associated with human leukocyte antigen HLA-DR3, and more severe disease has been associated with HLA-DQ/DR variants (*4*). Although most persons lack susceptibility, high nasal carriage rates in disease-endemic areas and living conditions associated with poverty further increase infection risk for susceptible persons because acquisition is facilitated by malnutrition, overcrowding, and poor sanitation (*5*).

Leprosy treatment is determined according to disease severity. The Ridley-Jopling system assesses lesion quantity, neurologic involvement, and bacterial load, and the current World Health Organization system simplifies this system to facilitate clinical classification, defining paucibacillary leprosy as <5 skin lesions and multibacillary leprosy as >6 lesions (*6*).

Combination drug regimens for 6–24 months are highly effective. Together with efforts of the World Health Organization toward eradication, combination therapy has dramatically reduced the prevalence to current levels from previously stable levels of 10–12 million in the 1960s–1980s (*7*). Typical regimens include dapsone and rifampin, and clofazimine is available in the United States by investigational new drug application for multibacillary disease.

Patients undergoing treatment must be monitored for immunologic complications, such as cell-mediated reversal reaction (type 1 reaction) or interferon-α–mediated erythema nodosum leprosum (type 2 reaction). Reversal reactions may be especially severe and require urgent immunosuppression to avoid neurologic and vascular complications.

Leprosy is extremely rare in the United States (150 annual cases). Because transmission by prolonged close contact is more common than by casual contact, it is likely that the infection in this patient may have been acquired during childhood in a disease-endemic area, which represents the upper limit of incubation time. However, rare cases have been reported among military members, which makes it difficult to exclude the question of acquisition during military service in disease-endemic areas (*8*–*10*). Therefore, in patients with geographically appropriate foreign service or prolonged travel history, leprosy must be considered in the differential diagnosis of progressive skin lesions, particularly when lesional anesthesia is present.
